# Genomic selection accuracy and bias using imputed genotypes on growth, welfare and fitness traits in two Pekin duck lines

**DOI:** 10.1186/s12864-026-12855-z

**Published:** 2026-04-22

**Authors:** Oswald Matika, Eirini Tarsani, Kiah McIntosh, Fasil G. Kebede, Andrea Talenti, Anne M. Rae, Andreas Kranis, Kellie A. Watson

**Affiliations:** 1 The Roslin Institute and Royal (Dick) School of Veterinary Studies, The University of Edinburgh, Easter Bush, Midlothian, EH25 9RG UK; 2 Department of Animal and Veterinary Sciences, Scotland’s Rural College (SRUC), Easter Bush, Midlothian, EH25 9RG UK; 3 Cherry Valley Farms (UK) Ltd, Cherry Valley House, Blossom Avenue, Humberston, Grimsby, DM36 4TQ UK; 4 Centre for Tropical Livestock Genetics and Health (CTLGH), Roslin Institute, University of Edinburgh, Easter Bush Campus, Midlothian, EH25 9RG UK

**Keywords:** Pekin ducks, Imputation, Genomic selection, Selection accuracy, Selection bias

## Abstract

**Background:**

The study explored the accuracy and biases of genomic selection in two commercial Pekin duck lines, focusing on their performance under real-world breeding practices. A dataset of 26 K duck records with phenotype and imputed genotype information (60 K chip) was analysed for growth, welfare, and primary feather length traits. Mixed linear models with relationship matrices from pedigree (BLUP) or markers (GBLUP) were used to estimate variance components and breeding values. We then assessed selection accuracies and biases to determine the most appropriate models.

**Results:**

Results showed high imputation accuracies of 0.93 for line A and 0.92 for line D. Heritability estimates from pedigree were generally higher than those from genomic markers. For example, juvenile weight (JW) heritability ranged from 0.22 in line A and 0.25 in line D using markers, to 0.39 and 0.50, respectively, using the pedigree. Slaughter body weight (BW) had similar trends. Gait heritability was low (0.07) using markers in both lines, while breast muscle depth (BD) also had lower estimates (0.15–0.16).

For line A, genomic prediction accuracies were higher with the G-matrix, especially for BW (r^2^=0.68-0.70) and JW with r^2^ of 0.49. Estimates for gait and foot pad dermatitis (FPD) improved using the G-matrix at 0.58 vs. 0.24 and 0.68 vs. 0.44, respectively, compared to pedigree information. Similar improvements were found for line D, with BD estimates improving from 0.50 to 0.71 using the G-matrix.

Line A showed minimal bias (0.01-0.19) with the G-matrix compared to 0.02-0.41 with the A-matrix; the highest bias was for JW. Line D had lower biases with the G-matrix (0.02-0.17) than in line A with markers, while higher biases were observed using pedigree (0.01-0.37).

**Conclusions:**

These findings indicate that all traits were heritable with higher prediction accuracies and lower biases using GBLUP compared to BLUP. This study demonstrates the effectiveness of GBLUP in improving prediction accuracy and reducing bias in selection traits of Pekin ducks, particularly for traits with low heritability.

## Background

The Pekin duck has been well documented for growth performance and meat quality traits [1–3]. In the past, poultry breeding programmes have relied on pedigree information usually gathered using single sires (ensuring the male parent is known) housed with a group of females, where artificial trap nest are used to identify the female parent. However, these are labour intensive, costly to manage and do not reflect normal poultry management practices. In addition, these may not be amenable to ever changing consumer and regulatory frameworks. The use of open pens for mating allowing many males and females in a single pen has been made possible by the use of parentage panels through Single Nucleotide polymorphism (SNP) genotyping. This can be achieved through the use of custom designed low density (LD) SNP genotype panels (300 to 1000 SNPs) or Medium-density (MD) SNP arrays (60k) and/or imputation. Parentage information is then used to construct pedigree information. Alternatively, the marker information can directly be used to construct relationship matrices to be used in the computation of breeding values, genomic selection (GS) and overall duck improvement programmes. GS was first proposed by Meuwissen, Hayes [4], using genotypes from a reference population to predict phenotypes when only marker information is available. Since then GS has been extended to combine both genotyped and ungenotyped animals when the pedigree is available in what is termed single-step GBLUB (ssGBLUP) [5]. Improved prediction accuracies have been reported in many species [6–8] when historic pedigreed data is available.

The use of G-matrices e.g. pair-wise identities by state (IBS) such as proposed by VanRaden [9] has several advantages over pedigree based A-matrix such as better estimating the Mendelian sampling term in close relatives [10–12], improving accuracies and efficiency [4, 12–14], offering the possibility of reducing pedigree-based inbreeding by using own rather than family information [11] and reducing generation interval by using younger animals for breeding [15]. In addition, genomic selection has lower selection biases estimates [16, 17] than pedigree-based selection practices.

Since genotyping using the MD SNP Chip is expensive for large flocks/herds, the strategy adopted for the current nucleus breeding flock was to use imputation from the parentage LD chip to MD Chip first proposed by [18] and used in layer chicken [19]. However, the accuracy of imputation is affected by many factors including the number of SNPs in the LD chip and their distribution along the genome, the size and connectedness of the reference population and the population genotyped with the LD Chip, marker allele frequencies etc [20–22].

Many studies in different species e.g. sheep [13, 23–25], cattle [26, 27], aquaculture [28, 29], pigs [30, 31] and chicken [19, 32] among other species have been published on the merits of genomic selection in livestock species, the few studies published for ducks which used real data had smaller sample sizes [14, 33–35] .

The traits considered in this current study include growth, primary feather length and low heritability traits for welfare such as gait as a proxy for leg health. Primary feather length (PRF) has been studied in poultry species for feather growth has a significant meaning in understanding adaptive evolution, physiology, and mating of avian species. They are also important for thermoregulation. GWAS results on primary feather length in ducks identified QTL with potential for effects on growth and maturity in ducks [36, 37]. The primary feathers of ducks have been reported to have economic value of their own when transformed into products such as badminton shuttles, feather pens and more recently into complex industrial products [38–40]. On farms, they can be used as an indication of maturity.

Genetic selection for increased body weights in chicken resulted in associated problems of locomotion (gait) [41], therefore it is imperative to measure gait score in any poultry breeding programme to mitigate any potential future welfare issues [42–44].

Foot-pad dermatitis (FPD), an ulceration of the skin of the foot in poultry [45], is associated with welfare issues [46–48] and in poultry has a high reported prevalence in European countries [49]. Scoring of footpads is used as an indicator to assess welfare in broiler production systems [50] and in ducks [51].

The aim of the current study is to investigate the genomic selection accuracies and biases estimates from two commercial Pekin duck lines reared under commercial breeding practices. The parentage genotype panel (Low density panel) captured as part of a genomic parentage programme across two lines of pedigree ducks was imputed to a medium density 60 K SNP panel (MD). We then estimated variance component parameters, genomic prediction accuracies and biases on 13 K records per line on growth, gait and primary feather length traits. Initially, data were analysed using mixed linear models with relationship matrices computed from pedigree (BLUP) or markers (GBLUP) to estimate the variance components and breeding values. After which, we assessed the predictive genomic performance using forward prediction by estimating the selection accuracies and selection biases.

## Materials and methods

### Animals

#### Management

Birds were reared according to standard commercial conditions using a starter feed for the first 14 days followed by a grower feed up to slaughter age. The light regime was implemented as follows: ducks had 23 h of light on the first day, decreasing by an hour a day until 18 h light was reached. The ducks then remained on 18 h of light until slaughter.

Data consisted of 3000 ducks sampled from two pure lines used in meat production, genotyped using the custom designed 60 K SNP array. The pedigree was reconstructed for a larger breeding population using a 427 custom built parentage SNP panel. For the two lines, all ducks with parentage SNP genotype were imputed to 60 K array genotype using the dense 60 K chip using Alphaimpute suite [52]. The two lines were genetically distinct, one selected for efficient growth (A) and the other for reproduction (D), only ducks with both imputed genotype and phenotype were retained for subsequent analyses. Data from Line A comprised of 13,645 and 13,020 for line D ducks with records for juvenile weight at 12 days (JW) and at slaughter: slaughter body weight (kg) (BW); ultrasonic measured breast depth (BD) obtained using an ultrasound probe placed parallel to the keel bone at the deepest part of the breast muscle with measurements taken from the keel bone to the top of the muscle; primary feather length (PRF); gait score (Gait) where a score of 1 represented very poor gait to 5 representing perfect gait, (for detailed gait score measurements on another duck line see Duggan, Rae [53]); average daily gain (ADG) and foot pad dermatitis score (FPD) was scored from 1 to 5, with 1 being no signs of FPD and 5 being highly ulcerated. The number of records per trait and full descriptive statistics of all traits are provided in Table 1.

**Table 1:** Descriptive and test for normality statistics for lines A and D

	Descriptive statistics							Tests for Normality		
	*N*	mean	STD	skewness	Kurtosis	CV	SE	Kolmogorov- Smirnov	Cramer- von Mises	Anderson- Darling
LINE A										
JW	13,610	567.15	86.27	-0.30	0.24	15.21	0.74	0.03	2.93	19.70
BW	13,640	4009.88	361.95	0.09	-0.23	9.03	3.10	0.01	0.61	3.94
BD	11,875	17.85	2.34	0.14	0.21	13.10	0.02	0.02	0.78	4.50
PRF	11,900	78.84	11.53	-0.76	1.38	14.63	0.11	0.13	21.97	123.24
GAIT	13,635	3.15	0.66	-0.14	0.41	20.82	0.01	0.31	276.19	1376.93
ADG	13,638	144.53	18.99	0.19	0.18	13.14	0.16	0.03	2.71	15.64
FPD	12,126	1.99	0.80	0.67	0.34	40.00	0.01	0.29	176.30	905.96
Line D										
JW	13,020	524.21	66.12	-0.17	0.26	12.61	0.06	0.02	1.02	5.96
BW	13,020	3204.60	294.20	0.11	-0.26	9.18	2.58	0.02	0.95	6.51
BD	11,241	21.56	2.88	-0.08	-0.13	13.35	0.03	0.02	0.65	4.14
PRF	11,277	105.12	11.85	-1.56	4.89	11.27	0.11	0.14	39.66	235.56
GAIT	13,012	3.10	0.61	0.15	0.87	19.57	0.01	0.35	341.41	1606.33
ADG	13,020	96.80	15.85	0.09	-0.23	16.37	0.14	0.02	0.69	4.07
FPD	10,693	1.94	0.66	0.24	-0.08	34.10	0.01	0.29	201.15	1030.73

*N *Number of records, *STD *Standard deviation, *SE* Standard error, *JW* Juvenile weight, *BW* Body weight, *PFR* Primary feather length, *ADG* Average daily gain to finish weight, *FPD* Foot pad dermatitis score

### Imputation

Approximately 17,000 to 18,000 animals per line were genotyped using a low-density (LD) SNP panel with 427 SNPs. For the training set used in imputation, 1,767 animals from line A and 1,536 animals from line D were genotyped with a medium-density (MD) 60 K SNP panel. The complete description of the imputation process for the GWAS, which utilized one of the duck lines, can be found in reference [54].

The imputation accuracies were calculated and validated using 200 ducks per line masking their genotype from the 60 K SNP chip array and after imputation, the same masked animals were compared to their imputed data to compute the accuracies.

All animals were imputed on the 60 K SNP chip up to 63,452 independent single nucleotide polymorphism (SNP) loci. After quality control (QC), SNPs with a minor allele frequency (MAF) less than 0.05 and those which did not meet the 1.00E-06 Hardy Weinberg Equilibrium (HWE) threshold were removed, remaining with a total of 47,156 and 46,297 SNPs for line A and D respectively, for further downstream analyses.

### Statistical analysis

Data were initially analysed in SAS 9.4 (SAS Institute Inc., Cary, NC, USA [55] to investigate the fixed effects for sex, hatching batch, age of dam and feeding pen at finishing. We also explored the interaction term between sex and hatch to give a fixed effect of “sexhatch”. The distributions of trait data values were checked for normality. Variance component analysis was conducted using linear mixed models fitting the environmental effects from the SAS models and fitting animal as random effect. The animal was fitted as random effect using either the available pedigree or the genomic relationship matrix.

Phenotypic data were pre-corrected in ASReml package [56] fitting the following models (1 and 2):

1 $$\mathrm{y}_\mathrm{ij}= \mu+ \mathrm{S}_\mathrm{i}+ \mathrm{A}_\mathrm{j}+\mathrm{e}_\mathrm{ij}$$

Where Y_ij_ is phenotype for JW, PRF, GAIT, FPD, S_i_=sexhatch and A_j_=age of dam.

2 $$\mathrm{y}_\mathrm{ijk} =\mu + \mathrm{S}_\mathrm{i} + \mathrm{A}_\mathrm{j} + \mathrm{Pk} + \mathrm{e}_\mathrm{ijk}$$

y_ijk_ is phenotype for BW and BD.

S=sexhatch, A = age of dam and P=feeding pen.

We also fitted BW as a covariate.

The resulting adjusted phenotypes or residuals (y*) were then fitted in a linear mixed model in the ASReml package [56], fitting the models (3 and 4):

3 $$\mathrm{y}* = \mu + \mathrm{Za} + \mathrm{e}$$

4 $$\mathrm{y}* = \mu + \mathrm{Zg} + \mathrm{e}$$

where y* is a vector of the adjusted phenotypic records, Z is a design matrix, a or g are vectors of either additive effects derived from the A-matrix (pedigree) or G-matrix (SNP markers) distributed respectively as N(0, σ_a_^2^A) or N(0,σ_g_^2^G), σ_a_^2^ or σ_g_^2^ are the corresponding additive genetic variances with A and G being the relationship matrices either derived from pedigree or the markers, and e is the vector of residuals. The G matrix in the present study was constructed using the methods proposed by VanRaden [9]. The marker data were visualised using Principal component analysis plots (PCA) performed using PLINK 1.9 [57]. available at https://www.cog-genomics.org/plink/1.9/ and R [58] and specifically the library qqman [59].

### Genomic prediction, accuracy and bias

Pre-corrected phenotypic data obtained using models from Eqs. 1 and 2 analysing separately duck data per line (A, D) from earlier generations (gen) being treated as reference (training set) and the validation sets obtained by masking the phenotype of all individuals from a given subsequent generation. This is the common practice in the commercial setting where juveniles (with no phenotype) will be selected using data from previously genotyped and phenotyped birds available. The numbers in each line are given in Table 1 and in each generation (GEN) in Table 2. For example, data for line A obtained generations 1 to 5 to predict birds in GEN 6. Genetic predictions for line D were for GEN 5 using data from GEN 1 to 4 as training set.

**Table 2:** The number of records in different generations (GEN) used in the genomic predictions for Line A and D

GEN	A	D
1	136	111
2	105	312
3	264	2780
4	4125	4932
5	4949	4885
6	4066	NA
Total	13,645	13,020

The genetic variance/covariance were estimated separately for each line using either pedigree (PEBV) or markers (GEBV) obtained from Eqs. 3 and 4 respectively. The reference ducks with phenotypes (non-masked) were then analysed by the model described above to predict the genomic breeding values (PGEBV) or with pedigree (PPEBV) predicted of the validation set whose phenotype were masked-phenotype individuals (i.e., in line A, 9579 ducks in generation 1 to 5, were used to predict 4066 ducks in generation 6 and in Line D, 8134 ducks from generation 1 to 4, to predict 4885 ducks in generation 5).

Pedigree or genomic prediction accuracies were calculated for each validation set (both within lines). Initially, the Pearson correlations of PGEBV with the adjusted phenotypes (*r*_gy_) were calculated and the accuracy (*r*_gg_) for each validation set was estimated by dividing *r*_gy_ by the square root of the heritability of each trait for that specific validation set (in our case GEN 1–5 for line A and 1–4 for line D) as given by Legarra et al. [60]:

$$Accuracy=\frac{r_{\widehat g\widehat y}}{\sqrt{h_y^2}}$$

Bias was measured as the regression of “True” EBV (estimated with phenotype) on Predicted EBV (Phenotype Masked) using both marker and pedigree information. The bias (˜b) is defined as the standardised regression coefficient of TBV on EBV as explained in [17, 61–63] and standardised bias was given by Lipschutz-Powell et al. [16] in a formula below:

$$\mathrm{Stnd}\;\mathrm{bias}\;=\;\left\{\begin{array}{l}1-\mathrm b\;\mathrm b<1\\1/\mathrm b\;-\;1\;\mathrm b\;\geq1\;\end{array}\right.$$

With equation `TBV = *b* * EBV`, if *b* is smaller than 1, then EBV is larger than TBV i.e. overestimated. If *b* is greater than 1, then EBV is smaller than TBV i.e. underestimated. However, the standardised *˜b* rescales and inverts the bias so it is from − 1 to 1 with negative being underestimation and positive being overestimation.

## Results

We evaluated the distributional properties of Line A data by calculating skewness and kurtosis coefficients (Table 1). The majority of traits (6 out of 7) displayed excellent adherence to normality, with coefficients ranging between − 0.5 and + 0.5. While PRF exhibited moderate negative skewness (-0.76) and positive kurtosis (+ 1.38), which are within the acceptable threshold of |2.0| for robust linear mixed model analysis. Similarly, six out of seven traits in line D (Table 1) exhibit adherence to normality, with skewness and kurtosis values firmly within the ± 0.5 range. This result underscores their highly symmetric and mesokurtic distributions, which allow the underlying assumptions of normality in our genetic evaluation model.

**Fig. 1:**
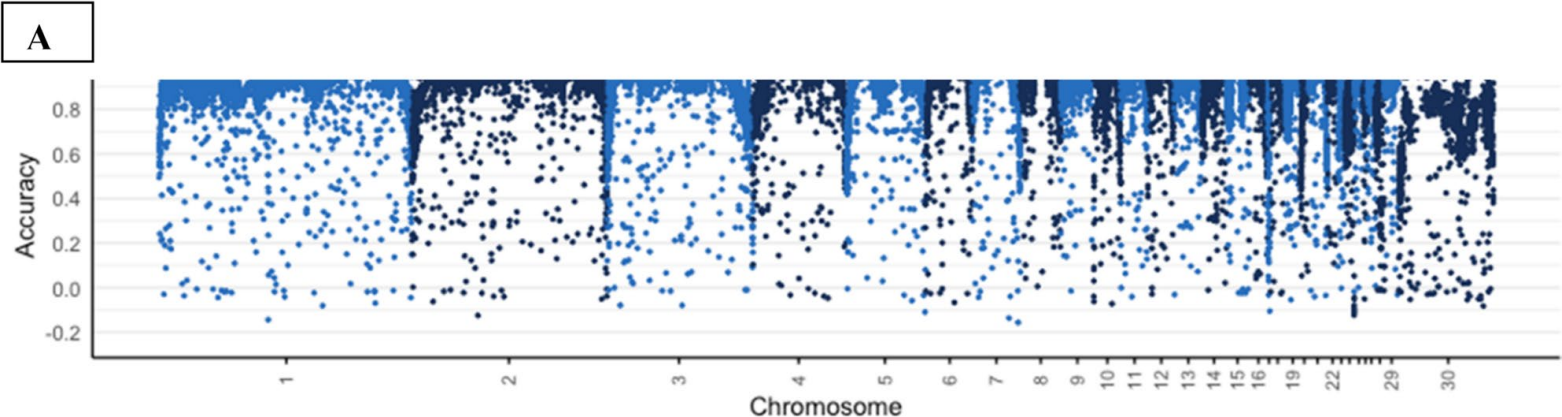
Duck whole genome imputation accuracies for lines A

**Fig. 2:**
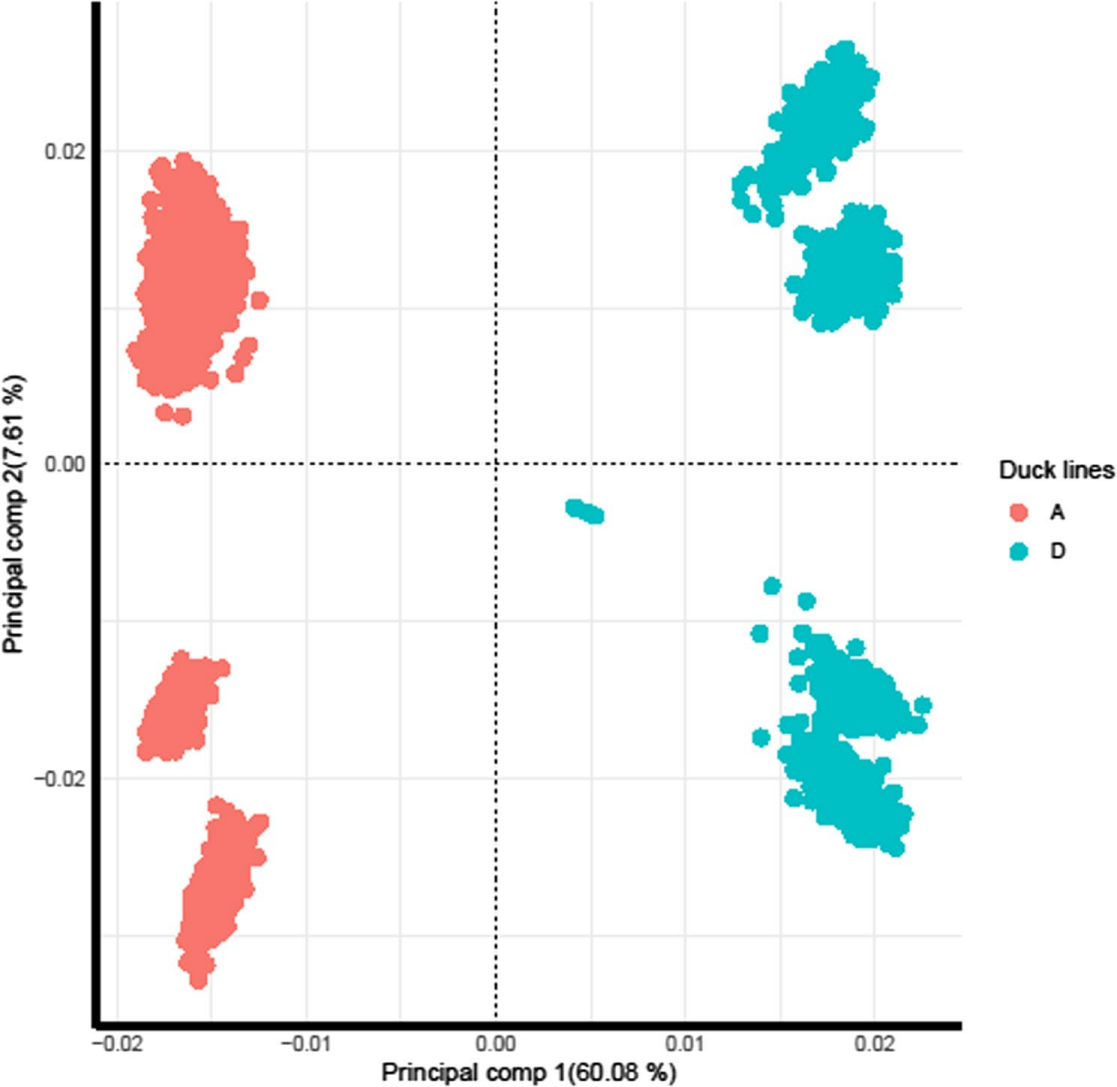
Principal component plot for 1782 ducks from line A and 1571 ducks from line D, using 60 K SNP genotype data

There were high imputation accuracies of 0.93 and 0.92 for lines A and D respectively. We observed lower accuracies at the telomere and on the sex chromosome, Z (see Fig. 1 for chromosomal distribution of accuracies for line A). The chromosomal accuracies for line D which followed a similar patten to line A was published by Tarsani et al. [54]. The first principal component (PC1) explained most of the variation (60.08%) with 7.61% explained by the second principal component (PC2). The PCA plot revealed that the two lines A and D were separated into distinct clusters by PC1. In addition, The PCA also identified distinct clusters within each line separated by PC2 (Fig. 2).

### Variance component analyses

All traits presented low to moderate estimates of heritability in either pedigree or marker information in both lines (Table 3). The estimates of heritability using the pedigree were generally higher than using genomic information in all traits considered in both lines. These ranged from 0.22 ± 0.01 for juvenile weight (JW) vs. 0.25 ± 0.01 in line A vs. line D using marker information to 0.39 ± 0.02 to 0.50 ± 0.02 using pedigree in line A vs. line D for slaughter body weight (BW) (Table 3). Very low estimates of heritability were recorded for gait 0.07 ± 0.01 using markers in both lines. BD had also lower estimates of 0.15–0.16 using markers.

**Table 3:** Heritability estimates (*h*^2^) for Duck lines A and D growth using pedigree (A-Matrix) and relationship matrices (G-Matrix) using all available records

	Line A			Line D		
	G-Matrix ( h^2^_G_)	A-Matrix ( h^2^_A_)	h^2^_G_/h^2^_A_	G-Matrix (h^2^_G_)	A-Matrix ( h^2^_A_)	h^2^_G_/h^2^_A_
JW	0.23 ± 0.01	0.41 ± 0.02	0.56	0.25 ± 0.01	0.46 ± 0.02	0.54
BW	0.33 ± 0.01	0.31 ± 0.02	1.06	0.37 ± 0.02	0.50 ± 0.02	0.74
BD	0.17 ± 0.01	0.22 ± 0.02	0.77	0.15 ± 0.01	0.21 ± 0.02	0.71
PRF	0.25 ± 0.01	0.31 ± 0.02	0.81	0.19 ± 0.01	0.26 ± 0.02	0.73
GAIT	0.07 ± 0.01	0.10 ± 0.01	0.70	0.07 ± 0.01	0.10 ± 0.01	0.70
ADG	0.17 ± 0.01	0.13 ± 0.01	1.31	0.22 ± 0.01	0.31 ± 0.02	0.71
FPD	0.25 ± 0.01	0.30 ± 0.02	0.83	0.21 ± 0.01	0.25 ± 0.02	0.84

## Genomic predictions

The genomic predictions were generally higher using the G-matrix than the A-matrix for line A (Table 4). The highest prediction was for body weights (*r*^*2*^ = 0.68–0.70) outside those of juvenile weight with *r*^*2*^ of 0.49. The estimates for gait and FPD were greatly improved by using the G-Matrix 0.58 vs. 0.24 and 0.68 vs. 0.44 respectively for markers vs. pedigree information.

**Table 4:** Genomic selection parameter estimates from Line A growth traits data obtained from generation 1 to 5 to predict generation 6 and selection bias using both A and G matrices in Asreml software with heritability estimates (*h*^2^) from 1–5 GEN records

	G_Matrix						A-Matrix					
Trait	h^2^	se	*r* ^2^	acc	b	˜b	h^2^	se	*r* ^2^	acc	b	˜b
JW	0.25	0.02	0.24	**0.49**	0.81	0.19	0.44	0.03	0.16	**0.24**	0.59	0.41
BW	0.32	0.02	0.39	**0.68**	1.02	-0.02	0.36	0.03	0.21	**0.35**	0.92	0.08
BD	0.2	0.02	0.22	**0.5**	0.89	0.11	0.23	0.02	0.13	**0.26**	0.72	0.28
PRF	0.24	0.02	0.34	**0.69**	0.99	0.01	0.31	0.03	0.22	**0.4**	0.94	0.06
GAIT	0.06	0.01	0.14	**0.58**	1.02	-0.02	0.08	0.01	0.07	**0.24**	0.75	0.25
ADG	0.15	0.01	0.25	**0.65**	1.1	-0.09	0.11	0.02	0.13	**0.4**	1.22	-0.18
FPD	0.26	0.02	0.34	**0.68**	0.99	0.01	0.29	0.03	0.23	**0.44**	0.98	0.02

Where Accuracy (acc)= corr(EBV_no_pheno_, TBV_with_pheno_ )/ √ (*h*^2^), b = reg.corf(EBV_no_pheno_, TBV_with_pheno_) standard bias (*˜b*)={1-b for b < 1, (1/*b*-1) for *b* > 1}

The same improvements for the G-Matrix vs. A-Matrix were observed in Line D (Table 5). The estimates for BD were similar in the two lines, however, the use of slaughter body weight greatly improved the G-Matrix estimates from 0.50 to 0.71 unlike in line A where they remained at 0.50.

**Table 5:** Genomic selection parameter estimates from Line D growth traits data obtained from generation 1 to 4 to predict generation 5 and selection bias using both A and G matrices in Asreml software with heritability estimates (*h*^2^) from 1–4 GEN records

Trait	G_Matrix						A-Matrix					
	h^2^	se	*r* ^2^	acc	b	˜b	h^2^	se	*r* ^2^	acc	b	˜b
JW	0.27	0.02	0.29	0.55	0.91	0.09	0.46	0.03	0.26	0.38	0.83	0.17
BW	0.4	0.02	0.44	0.69	0.97	0.03	0.55	0.03	0.29	0.39	0.92	0.08
BD	0.17	0.02	0.21	0.5	0.83	0.17	0.21	0.02	0.13	0.28	0.63	0.37
PRF	0.25	0.03	0.29	0.58	0.98	0.02	0.18	0.02	0.19	0.44	1.01	-0.01
GAIT	0.06	0.02	0.13	0.51	0.92	0.08	0.1	0.02	0.08	0.26	0.92	0.08
ADG	0.28	0.03	0.3	0.56	0.93	0.07	0.34	0.03	0.12	0.21	0.72	0.28
FPD	0.21	0.05	0.33	0.73	1.08	-0.07	0.23	0.03	0.22	0.47	0.99	0.01

Where Accuracy (acc)= corr(EBV_nopheno_, TBV_withpheno_ )/ √ (*h*^2^), b = reg.corf(EBV_nopheno_, TBV_withpheno_) standard bias (*˜b)*={1-b for b < 1, (1/b-1) for b > 1}

The biases in line A were minimal (0.01–0.19) when using the G-Matrix compared to 0.02–0.41 those when using the A-Matrix. The highest observed bias was for JW followed by BD for the G-matrix whereas using the A-matrix higher biases were observed in many traits (JW, BW, BD, gait etc.), see Table 4 for more details. The biases for line D were generally lower for G-matrix (0.02–0.17 vs. 0.00–0.19) than those observed in line A when using markers (Table 5). However, higher biases were observed using the pedigree (0.01–0.37).

## Discussion

The main objective of the present study was to explore forward predictive power of GS using a large dataset on accuracies and biases of the estimated breeding values on selection candidates (without own records) on both growth, welfare and fitness traits in Pekin ducks. In genomic selection, variance components of linear mixed models are ideally estimated with all available data to avoid bias. However, due to computational limitations in handling large datasets or complex models, we first fitted fixed effects and then used the residuals as phenotypes. This approach addresses both hardware and software constraints effectively. Notably, the heritability estimates obtained were within expected ranges, validating this method. Additionally, in scenarios such as across-country studies [64, 65] or when combining data from private companies, deregressed EBVs or pre-corrected phenotypes are typically used since sharing raw data can be sensitive.

Our current study is unique in that it used two different lines, over 13k records per line and seven diverse traits using imputed data to study variance components and genomic selection accuracies and biases in a commercial setting using real field data. All traits were heritable, demonstrating genetic influence across growth, welfare and fitness traits. Multidimensional plots revealed no discernible population structure, indicating a uniform genetic background within the lines. However, PCA plot shows two distinct lines from PC1 (Fig. 2).

In our study, we explored a range of traits with varying heritability estimates, from low for gait to moderate for primary feather patterns and foot pads, and generally high estimates for production traits when using the A-matrix. We observed in general, lower heritability estimates when we used the G-Matrix as opposed to the A-Matrix. The estimates of heritability were very different between the two lines. The proportion of heritability estimates recovered by the markers was also different in the two lines as we observed an overestimation in Line A, of heritability estimates in BW and ADG. However, this was not the same case for the same traits in line D, although GWAS identified a large QTL [54]. The reasons for missing heritability are many and varied and well-studied in human height and diseases [66, 67] with solutions including among others increasing sample sizes, using more rare variants and structural variations [68] not captured in SNP-chips used to create the G-Matrix [69]. Some attribute the missing heritability to rare variants that can be captured by whole sequence data [70] and use of haplotypes [71]. Génin [69] in a review, believed that the “missing heritability problem” is an ill-posed problem and may be due to complexity of the biology of traits rather than statistical methods of partition heritability estimates. Golan et al. [72] attributed the problem of missing heritability to the use of common variants and that restricted maximum likelihood (REML) estimation underestimated the fraction of heritability due to common variation considerably. Instead, they propose phenotype correlation-genotype correlation (PCGC) regression. In their study of six diseases, they estimated the proportion of heritability due to common variants from 41% to 68% (mean 60%) using their method. Others attribute the lack of concordance between markers and traditional methods to lack of capturing additive epigenetic variances [66, 73].

Hu et al. [74] reported heritability of primary feather length of 0.37 ± 0.04 in males and 0.14 ± 0.02 in females. They also reported high genetic correlations of PRF with body weights at 10 and 18 weeks of age implying that selecting for PRF will have indirect effects on growth.

The current estimates of heritability for gait were low (0.06 ± 0.06 and 0.12 ± 0.06) and similar to those reported by Duggan et al. [53] in Pekin flocks albeit in different years, different lines and with different numbers of records making this trait a good candidate for genomic selection.

The reported genetic component to FPD in different lines and breeds [49, 75, 76] ranged from low [76] to medium [49]. Kapell et al. [75] reported in broilers heritability estimates of 0.18 ± 0.02 to 0.24 ± 0.02 using the pedigree and concluded that selection against FPD in a highly biosecure environment could improve the genetic merit for birds reared under commercial conditions since there was a high genetic correlation (0.78–0.82) between FPD in the pedigree and in the sib-tested environments. Ask [76] report heritability estimates for FPD in two broiler flocks of 0.21 ± 0.03 and a genetic correlation with BW of -0.51 ± 12.

We obtained higher prediction accuracy estimates when using the G-Matrix as opposed to the A-matrix and on the other hand, lower biases across the two lines irrespective of the large QTLs identified in Line D. The most significant enhancement in selection accuracy estimates was observed with gait score, used as a proxy for leg health. This aligns with expectations, as genomic selection is particularly effective for traits that are difficult to measure. Utilizing markers leads to higher predictive accuracies, which consequently results in greater genetic gains.

For GS to work, one constantly needs animals genotyped with an MD chip so that the link between generations is maintained. This requirement is less in this flock since all juveniles are genotyped for parentage ascertainment. In many previous studies cross-validation was used to calculate accuracies in sheep [13], in pigs [22] and in ducks [77, 78] among many others in poultry and dairy and beef cattle. The common thread is that usually, the animals are below a thousand and the genotyped animals per generation are often not sufficient to predict across generations. However, in the current study we had around 8 K animals (reference set) to predict over 4k in the last generation (selection candidates). Other methodologies such as LR, have been proposed to evaluate the cross-validation tools using “partial” and “whole” data [63], however, we consider this method inappropriate in the current study, since we have fewer records in earlier generations and the aim was to inform breeding practices in a commercial breeding nucleus flock where they are interested in forward predictive power of GS.

In some studies, they found that using the single step methods overestimated variance components [79]. In sheep, Macedo et al. [6] found that including additional information of meta founders reduced biases for milk yield but not in some traits but also observed over-dispersion when using single-step GBLUP. They also concluded that the use of additional genomic information increased the accuracies of GEBV for young rams in their population. However, in a review, Misztal et al. [5] discussed conditions which need to be satisfied to make ssGBLUP unbiased and noted that is now the method of choice in large commercial breeding programmes (pigs, chicken etc.) other than dairy cattle. Muir [80] showed that, in comparison of traditional BLUP and GBLUP, with both genotypes and phenotypes collected in many generations, the better was the accuracy and persistency of accuracy based on GEBV. They also showed that GEBV excelled for traits of low heritability regardless of initial equilibrium conditions and low persistence of genomic prediction under selection. However, in the current flock, all ducks are genotyped to determine parentage, making ssGBLUP unnecessary. In the current breeding programme, some of the conditions of persistence of linkage of markers across future generations will be mitigated with the addition of new animals genotyped with MD SNP chip. The traits with low heritability estimates such as gait will have the greatest benefit from use of GBLUP and genomic prediction.

## Conclusion

Our results are from a unique large dataset of almost 26 K ducks with about 13 K records per line in a species where there is limited published data on both growth and welfare traits. We also demonstrated reasonable accuracies using imputed genotype data. For all traits we analysed, we observed generally higher heritability estimates from the pedigree than genomic data, with higher prediction accuracies and lower biases when using GBLUP as opposed to traditional BLUP. It is important to note we observed higher accuracies in welfare traits which tend to have lower heritability estimates, hence the chance to make faster genetic progress in these hard to measure traits.

## Data Availability

The datasets produced or analysed during this study are not publicly accessible due to the commercial confidentiality of the breeding scheme. However, they can be obtained from Cherry Valley Ltd by making a reasonable request and signing a confidentiality agreement. Please contact Anne Rae ( [anne.rae@cherryvalley.co.uk] (mailto: anne.rae@cherryvalley.co.uk) ) for more information.
